# Adenylate kinase 5, a novel genetic risk factor for Alzheimer’s disease, regulates microglial inflammatory activation

**DOI:** 10.1186/s13041-025-01257-z

**Published:** 2025-11-26

**Authors:** Won Jae Seong, Sang Joon An, Jungsoo Gim, Deepak Prasad Gupta, Junyoung Park, Sarang Kang, Kun Ho Lee, Gyun Jee Song

**Affiliations:** 1 Department of Medicine, College of Medicine, Catholic Kwandong University, Gangneung, Gangwon-do Republic of Korea; 2 Translational Brain Research Center, International St. Mary’s Hospital, Catholic Kwandong University, Incheon, Republic of Korea; 3 Department of Neurology, College of Medicine, International St. Mary’s Hospital, Catholic Kwandong University, Incheon, Republic of Korea; 4 Gwangju Alzheimer’s and Related Dementia Cohort Research Center, Chosun University, Gwangju, Republic of Korea; 5 Department of Biomedical Science, BK21 FOUR Educational Research Group for Age-Associated Disorder Control Technology, Chosun University, Gwangju, Republic of Korea; 6 Korea Brain Research Institute, Daegu, Republic of Korea; 7 Department of Neurology and Neurological Sciences, Stanford University, Stanford, CA USA

**Keywords:** Adenylate kinase 5, Alzheimer’s disease, Microglia, Neuroinflammation, Genome-wide association study

## Abstract

Alzheimer’s disease (AD) is a progressive neurodegenerative disorder characterized by memory loss, cognitive decline, and neuroinflammation, primarily mediated by microglia. In this study, we investigate the role of adenylate kinase 5 (AK5) in microglial function and its association with AD-related pathology. Analysis of brain tissues from AD patients and AD model mice revealed a significant reduction in AK5 expression. In vitro knockdown of AK5 in microglial cells attenuated lipopolysaccharide-induced pro-inflammatory responses, including decreased nitric oxide and tumor necrosis factor-alpha production, while enhancing phagocytic activity. Moreover, AK5 silencing induced metabolic reprogramming, evidenced by reduced lipid droplet accumulation and adipose triglyceride lipase mRNA levels, alongside increased farnesoid X receptor mRNA expression. Genome-wide association studies further identified two AK5 single nucleotide polymorphisms (SNPs), rs59556669 and rs75224576, significantly associated with hippocampal and amygdala atrophy as well as increased AD risk. Notably, these SNPs were not in linkage disequilibrium with the apolipoprotein E (APOE) locus, suggesting that AK5 may represent an independent genetic risk factor for AD. Collectively, our findings identify AK5 as a key regulator of microglial immune and metabolic function. The presence of AK5 variants may contribute to AD susceptibility, and AK5 expression or genetic status could serve as a potential biomarker for early risk assessment. Further exploration of AK5-targeted interventions may provide new therapeutic avenues for AD prevention or treatment.

**Supplementary Information:**

The online version contains supplementary material available at 10.1186/s13041-025-01257-z.

## Background

Alzheimer’s disease (AD) is the most common chronic and progressive form of senile dementia globally, clinically characterized by memory deficits, cognitive impairment, and intellectual decline [1]. The pathophysiology of AD prominently involves neuronal loss, oxidative stress, and neuroinflammation, predominantly mediated by microglia [2, 3]. Microglia, the brain’s resident immune cells, play a critical role in the pathogenesis of neurological diseases, including AD [2, 4]. These immune cells display dynamic responses to environmental stimuli, transitioning from a quiescent state to a phagocytic phenotype in response to factors such as amyloid β accumulation and neuronal degeneration [5]. Modulating microglial phagocytic activity within specific therapeutic windows may offer a viable strategy for AD treatment [2, 4, 6].

AD poses a formidable challenge due to the lack of effective therapies to date [7]. With the increasing prevalence of dementia in the aging population [8, 9], there is an urgent need for novel pharmacological targets. Genetic research is pivotal in identifying these targets, which is essential for the development of effective AD treatments [10, 11]. Recent large-scale genome-wide association studies, including the work by Bellenguez et al. [11], have identified 75 AD risk loci, 42 of which were novel, highlighting not only classical amyloid/tau pathways but also the involvement of microglial functions. Transcriptomic analyses, including a report by Annese et al. [12], have identified 2064 genes dysregulated in hippocampal regions of AD patients through ribonucleic acid (RNA) sequencing. However, the functional implications of these genetic findings and their potential as therapeutic targets remain incompletely understood.

The adenylate kinase (AK) family of isoenzymes plays a pivotal role in nucleotide metabolism by facilitating phosphate transfer from adenosine triphosphate (ATP) to adenosine monophosphate (AMP), resulting in the generation of two adenosine diphosphate (ADP) molecules, or transferring a phosphate group from two ADP molecules to form one ATP and one AMP molecule [13]. Regulation of intracellular AMP levels is essential for various cellular processes [14–16]. Given their ubiquitous nature, AK isoenzymes may present therapeutic targets for certain cancers or serve as candidates for novel antibiotics [13, 17]. Among the AK isoenzyme family, AK5 merits particular attention due to its abundance in the brain, particularly in the cerebral cortex and hippocampus [18], suggesting a brain-specific function and potential as a target for the treatment of neurological diseases.

Together, these considerations led us to hypothesize that AK5, as a brain-enriched metabolic enzyme, plays a regulatory role in microglial metabolism and inflammation, and represents a novel genetic contributor to AD pathogenesis.

## Methods

### Cell culture

Mouse primary mixed glial cell (MGC) cultures were derived from the brains of 3-day-old C57BL/6 mice. Brains were dissected, homogenized, and mechanically dissociated using a nylon mesh. MGCs were seeded in poly-l-lysine-coated flasks with Dulbecco’s Modified Eagle Medium (DMEM, Gibco, Grand Island, NY, USA) supplemented with 10% fetal bovine serum (FBS), 100 U/mL penicillin, and 100 μg/mL streptomycin (Gibco) and incubated at 37 °C in a humidified 5% CO_2_ atmosphere. The culture medium was replaced after 5 days and then every 3 days. After 14 days, MGCs were harvested by trypsinization as previously described [19] and plated in the same media for experimental use. The BV-2 immortalized murine microglial cell line was cultured in DMEM with 5% heat-inactivated FBS and 50 μg/mL gentamicin (Gibco) at 37 °C in 5% CO_2_.

### Identification of key kinases for glial activation using kinase siRNA library

To identify key kinases for glial activation, we previously performed a small interfering RNA (siRNA) screening using a library of 623 mouse kinases (MISSION siRNA Mouse Kinase Panel, Sigma-Aldrich, St. Louis, MO, USA). The screening was optimized for MGCs and involved transfection using Lipofectamine RNAiMAX (Thermo Fisher Scientific, Waltham, MA, USA). Following transfection, multiple glial functions were assessed, including anti-inflammatory capacity via the Griess reagent assay, cytotoxicity via the MTT reagent assay (Sigma-Aldrich), and migration capacity using the IncuCyte ZOOM Live-Cell Imaging system (Essen BioScience, Ann Arbor, MI, USA) as previously described [20]. This multiplexed siRNA screening identified candidate kinases regulating both inflammation and wound-healing processes in glial cells (Supplementary Table 1).

### Assessment of nitric oxide (NO) production

MGCs and BV-2 cells were plated in 96-well plates at a density of 4 × 10^4^ cells per well. MGCs were treated with lipopolysaccharide (LPS, Sigma-Aldrich, 1 μg/mL for 48 h) or with a combination of LPS (LPS 1 μg/mL) and interferon-γ (IFN-γ, 50 U/mL, R&D Systems, Minneapolis, MN, USA) for 24 h. BV-2 cells were treated with LPS (100 ng/mL) for 24 h. NO production was quantified using the Griess assay, as described previously [21]. Briefly, 50 μL of the cell culture supernatant was collected after 24 h of incubation and mixed with an equal volume of Griess reagent (0.1% *N*-1-Naphthyl ethylenediamine dihydrochloride and 1% sulfanilamide in 5% phosphoric acid) in a 96-well microtiter plate. Absorbance was measured at 540 nm using GloMax® Microplate Reader (Promega, Madison, WI, USA), and sodium nitrite was used to generate the standard curve for NO concentration calculation.

### Assessment of cell viability and cell migration

Both BV-2 cells and MGCs (4 × 10^4^ cells/well in 96-well plates) were used to assess cell viability via the MTT assay, following established protocols [19]. After 24 h of treatment with LPS or a combination of LPS and IFN-γ, the culture media was aspirated, and MTT solution (0.5 mg/mL in phosphate-buffered saline) was added to the cells, followed by incubation at 37 °C for 2 h in a 5% CO_2_ environment. The resulting formazan crystals were solubilized in dimethyl sulfoxide (DMSO), and absorbance at 570 nm was measured using a GloMax® Microplate Reader (Promega, Madison, WI, USA). MGCs (4 × 10^4^ cells/well in 96-well plates) were used to assess cell migration via a wound healing assay, as previously reported [21].

### Conventional and real-time reverse transcription polymerase chain reaction

Total RNA was extracted from treated cells and brain tissues using TRIzol reagent (Invitrogen, Carlsbad, CA, USA). Reverse transcription was performed with Superscript II reverse transcriptase (Invitrogen) and oligo(dT) primers. Conventional polymerase chain reaction (PCR) amplification was carried out using specific primer sets with annealing temperatures of 55–60 °C and 25–32 cycles on a C1000 Touch Thermal Cycler (Bio-Rad, Hercules, CA, USA). PCR products, stained with ethidium bromide, were electrophoresed on 2% agarose gels and visualized under ultraviolet light. Real-time PCR was conducted using the One Step SYBR PrimeScript RT-PCR Kit (Takara Bio, Otsu, Shiga, Japan) according to the manufacturer's instructions, with detection performed on the ABI Prism 7000 Sequence Detection System (Applied Biosystems, CA, USA). Glyceraldehyde 3-phosphate dehydrogenase (GAPDH) was used as the internal control. Primer sequences were designed based on published complementary DNA sequences (Table 1).

**Table 1:** Primer sequences for PCR

Gene	F/R	Primer sequence	Gene ID
GAPDH	F	5′-CTCATGACCACAGTCCATGC-3′	NM_008084.2
	R	5′-TTCAGCTCTGGGATGACCTT-3′	
AK5	F	5′-ACTAGACCTCGGCCCAAAAT-3′	NM_001081277.3
	R	5′-TCCATTTTCTGTTGCTGCTG-3′	
TNF-α	F	5′-CCAACGGCATGGATCTCAAAGACA-3′	NM_013693.3
	R	5′-AGATAGCAAATCGGCTGACGGTGT-3′	
IL-1β	F	5′-CTTTGAAGAAGAGCCCATCC-3′	NM_008361.3
	R	5′-TTTGTCGTTGCTTGGTTCTC-3′	
ATGL	F	5′-CAC TTT AGC TCC AAG GAT GA-3′	NM_001163689
	R	5′-TGG TTC AGT AGG CCA TTC CT-3′	
FXR	F	5′-TCCGGACATTCAACCATCAC-3′	NM_001385711.1
	R	5′-TCACTGCACATCCCAGATCTC-3′	

### Western blotting analysis

Cells or brain tissues were lysed in 300 μL of lysis buffer (150 mM sodium chloride, 1% Triton X-100, 1% sodium deoxycholate, 0.1% sodium dodecyl sulfate (SDS), 50 mM Tris–HCl (pH 7.5), 2 mM Ethylenediaminetetraacetic Acid) supplemented with Halt™ protease and phosphatase inhibitor cocktail (1×) (Thermo Fisher Scientific). Brain tissues were individually homogenized and then centrifuged at 13,400×*g* at 4 °C for 15 min. Protein concentration was determined using the Pierce BCA protein assay kit (Thermo Fisher Scientific), with bovine serum albumin as the standard. Equal amounts of protein (20–30 μg per sample) were separated using 12% sodium dodecyl sulfate–polyacrylamide gel electrophoresis (SDS–PAGE) and transferred to polyvinylidene fluoride (PVDF) membranes (Bio-Rad, Hercules, CA, USA) via the semi-dry electroblotting method. Membranes were blocked with 5% skim milk and incubated sequentially with primary antibodies against AK5 (1:1000, Rabbit, #12510-2-AP, Proteintech, Rosemont, IL, USA), BDNF (1:1000, Rabbit, ab108319, Abcam, Cambridge, MA, USA) or β-actin (1:4000, Mouse, MA5-15739, Thermo Fisher Scientific), followed by chemiluminescence detection using an enhanced chemiluminescence (ECL) kit (Thermo Fisher Scientific) and Amersham ImageQuant 800 imager (GE Healthcare, Pittsburgh, PA).

### Small interfering ribonucleic acid-mediated knockdown of the AK5 gene

Cells were transfected with a pool of siRNAs targeting *AK5* (siAK5) using Lipofectamine™ RNAiMAX according to the manufacturer’s protocol. The cells were harvested and used for experiments at least 48 h post-transfection. The siRNA sequences were as follows: siAK5-#1, 5′-GUGUCGUUCUGGAGCUGCUTT-3′, siAK5-#2, 5′-CACACUUGGUGAUCUGCAUTT-3′, and siAK5-#3, 5′-CAGCGGACACCAUGACUAATT-3′. A non-targeting control siRNA (siCon) was purchased from Genolution Pharmaceuticals (Seoul, South Korea) with the sequence: siCon—5′-CCUCGUGCCGUUCCAUCAGGUAGUU-3′.

### Phagocytosis assay utilizing zymosan and amyloid β

The phagocytosis assessed using pH-sensitive fluorescent zymosan bioparticles derived from *Saccharomyces cerevisiae* (pHrodo™ Red dye conjugates, Life Technologies, Carlsbad, CA, USA). BV-2 cells were seeded at a density of 5 × 10^4^ cells/well on poly-d-lysine-coated glass coverslips (12 mm diameter) and cultured for 48 h [22]. Cells were then treated with zymosan in serum-free DMEM for 3 h and examined under a fluorescence microscope (Leica Microsystems, DM2500, Wetzlar, Germany) using the red channel (excitation 560 nm, emission 585–600 nm). For amyloid β phagocytosis, fluorescein isothiocyanate (FITC)-Aβ_1–42_ (Bachem, Bubendorf, Switzerland) was aggregated for 24 h at 4 °C and added to the cells at a final concentration of 1 µM. After a 2-h incubation, cells were washed with fresh DMEM to remove extracellular Aβ. To further assess clearance after phagocytosis, cells were incubated with FITC-Aβ for 1 h, washed, and then cultured in full medium for an additional 1 h or 5 h before fluorescence measurements. The residual intracellular FITC-Aβ fluorescence was quantified at excitation/emission 485/538 nm using a NanoXpress system (Molecular Devices, San Jose, CA, USA). Intracellular fluorescence signals from zymosan (pHrodo Red) or FITC-Aβ were quantified using MetaXpress software (Molecular Devices) and normalized to control values.

### Lipid droplets staining

BV-2 cells were transfected with siRNA targeting the *AK5* gene and incubated for 48 h. Cells were then stained with BODIPY (1:2000 from 1 mg/mL stock solution in DMSO; Invitrogen Molecular Probes, Eugene, OR, USA) for 3 h at 37 °C. Fluorescence was measured at excitation/emission 485 nm and 538 nm using a NanoXpress system. Intracellular lipid droplets and fluorescent puncta were quantified using MetaXpress software.

### Animals

All animal experiments were conducted in accordance with the guidelines approved by the Catholic Kwandong University Institutional Animal Care and Use Committee, International Saint Mary’s Hospital (Approval No. CKU 2020-012). 5xFAD transgenic mice (B6SJL-Tg [APPSwF1Lon, PSEN1M146L × *L286V*] 6799Vas/Mmjax) were obtained from the Jackson Laboratory (Bar Harbor, ME, USA). Male wild-type (WT) and 5xFAD mice, provided by the Korea Brain Research Institute (Republic of Korea), were used as control and AD model mice, respectively. Genotyping of all mice was performed via PCR on genomic DNA extracted from tail biopsies.

Mice were housed under controlled conditions: humidity of 40–60%, temperature of 20–26 °C, and ventilation refreshed 15–16 times per hour. Lighting was set at 150–300 lx during a 12-h light cycle, followed by complete darkness during the 12-h-dark cycle. All animals were provided with food and water ad libitum. All experiments were conducted under the approved animal facility conditions of Catholic Kwandong University, International Saint Mary’s Hospital, Republic of Korea.

### Immunofluorescence staining of brain tissues

Mice were anesthetized and perfused intracardially with 0.9% saline. Brains were collected and post-fixed for 24 h in 4% paraformaldehyde. Coronal brain sections (20-μm thickness) were obtained using a CM3050S microtome (Leica Microsystems). Sections were permeabilized with 0.3% Triton X-100 in phosphate-buffered saline (PBST) and blocked with 0.3% normal donkey serum and 1% bovine serum albumin for 60 min at room temperature. Tissue sections were then incubated with following primary antibodies: ionized calcium-binding adaptor molecule 1 (Iba1, microglia marker, 1:200, goat,, NB100-1028, Novus Biologicals, Centennial, CO, USA), AK5 (1:100, rabbit, 12510-2-AP, Proteintech), or NeuN (neuron marker, 1:1000, rabbit, ABN78, Millipore, Burlington, MA, USA), as described previously [20]. Sections were counterstained with 4′,6-diamidino-2-phenylindole (DAPI) (Invitrogen Molecular Probes).

### Study participants for genetic studies

The study sample was selected from 21,382 participants aged 55–90 years in the Gwangju Alzheimer’s and Related Dementia cohort study in Gwangju, South Korea. The sample included 1110 individuals with AD dementia and 1172 cognitively normal age-matched individuals. A precise diagnosis, MR images and genomic data was available from all participants. The genotyping and quality control procedures have been described in a previous report [23]. Additionally, the association was studied in a subset of individuals who underwent brain imaging, as detailed in Table 2. The protocols for magnetic resonance imaging (MRI) and amyloid positron emission tomography (PET) imaging, including the quantitative analysis methods, have been described in a previous report [24]. Cognitively normal participants had no evidence of neurological disease or impairment in cognitive abilities or activities of daily living, as assessed by the Korean Mini-Mental Status Exam (K-MMSE) and other neuropsychological tests. Study protocols were approved by the institutional review board of Chosun University Hospital, and all participants, or the legal guardians of cognitively impaired individuals, provided informed consent before participation in the study (CHOSUN 2013-12-018-070). To identify associations within the *AK5* gene, we employed summary statistics from previous Genome-Wide Association Study (GWAS) on AD [23] and generated a regional association plot using LocusZoom (v1.4). The *AK5* SNPs showing genome-wide significant (GWS) association with AD risk were further evaluated in subjects with MRI and amyloid PET traits.

**Table 2:** Summary of study population

	NC	MCI	AD	*p* value
	N = 416	N = 398	N = 121	
Age	73.12	73.66	76.28	ns
Amyg (left)	1525 ± 227	1441 ± 249	1412 ± 298	< 0.0001
Amyg (Right)	1584 ± 213	1500 ± 281	1447 ± 290	< 0.0001
Hippo (left)	3857 ± 499	3603 ± 580	3612 ± 629	< 0.0001
Hippo (Right)	4115 ± 508	3822 ± 631	3793 ± 692	< 0.0001

*NC* normal cognition control, *MCI* mild cognitive impairment, *AD* Alzheimer’s disease, *Amyg* amygdala, *Hippo* Hippocampus, *ns* not significant

### Quantification and statistical analyses

Statistical analysis was performed using either an unpaired two-tailed Student’s *t-test* or a two-way ANOVA with Tukey’s multiple-comparisons test in Prism (version 10.2.3, GraphPad Software, San Diego, CA, USA). Differences with *p* values < 0.05 were considered statistically significant. For the immunohistochemical analysis, 5–6 microscopic images were chosen randomly for statistical analysis. For the measurement of immunofluorescence or western blot band intensities, the area of the entire image or each band was selected, and the mean intensity was measured using ImageJ software (version 1.54, National Institutes of Health, Bethesda, MD, USA).

## Results

### Reduction of AK5 expression in brain tissues of patients with AD

A multiplexed kinome-wide siRNA screen has previously identified key kinase signaling pathways regulating inflammatory phenotypes in glial cells [20]. Using a similar screening strategy, we performed NO production and cell migration assays to identify kinases exhibiting both anti-inflammatory and pro-migratory properties. Of the 623 kinases screened, 49 were found to modulate NO production while enhancing cell migration, as listed in Supplementary Table 1. Among these, siRNAs targeting 15 kinases demonstrated potent anti-inflammatory effects and promoted wound healing. We subsequently focused on adenylate kinase 5 (AK5) to explore its role in neuroinflammation and Alzheimer’s disease pathogenesis.

**Fig. 1:**
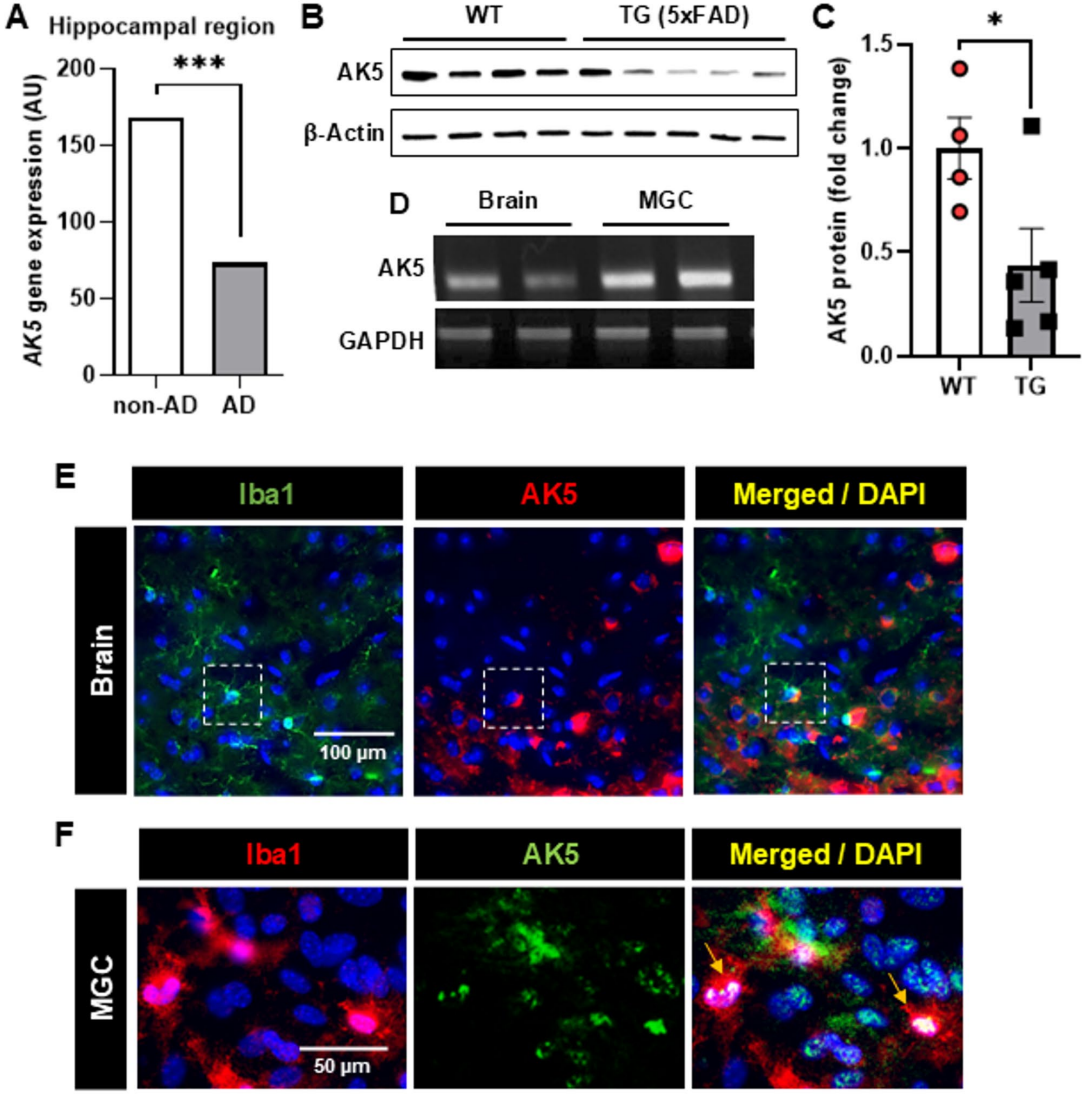
Reduced *AK5* expression in AD brain tissues and its localization in microglia. **A** *AK5* gene expression in the hippocampal region of patients with AD compared with non-AD controls. Data represent the average values from the dataset published in Annese et al. [12]. (***p < 0.001) **B** Representative western blot image showing AK5 and β-actin protein levels isolated from whole brain tissues of wild-type (WT) mice and AD transgenic (TG) mice (5xFAD). **C** Quantification of AK5 protein expression is shown as fold change normalized to β-actin. *p < 0.05 vs. WT, based on an unpaired two-tailed Student’s *t-test*; n = 4 for WT and n = 5 for the TG group; data are presented as mean ± standard error of the mean (SEM). **D** Representative image showing AK5 mRNA expression in whole brain tissues of WT mice, mixed glial cells (MGC) from WT mice. **E** Representative immunostaining images showing AK5 (red) expression co-localized with Iba1 (green)-positive microglia in the cortex of mouse brain tissue. Nuclei were stained with DAPI (blue). Microglia showing co-localization with AK5 are highlighted in the magnified images, as indicated by the dotted area. Scale bar = 100 μm.** F** Representative immunostaining images showing AK5 (green) expression co-localized with Iba1 (red)-positive microglia in mouse glial cells (MGCs). Nuclei were stained with DAPI (blue). Microglia showing co-localization with AK5 are highlighted with arrows. Scale bar = 50 μm

To investigate AK5 expression in the brains of patients with AD, we initially analyzed an open dataset of AD brain transcription profiles. A significant decrease in AK5 mRNA expression was observed in hippocampal tissues of patients with AD compared with non-AD controls (Fig. 1A). This reduction was further validated in 8-month-old AD model mice using western blot analysis, which demonstrated a marked decrease in AK5 protein levels in AD mice compared with WT controls (Fig. 1B, C). Previous research has noted AK5 expression primarily in neurons [18]. To investigate AK5 expression in glia, we conducted RT-PCR. Our findings revealed robust AK5 expression in brain tissue and mixed glial cultures (MGCs) (Fig. 1D). Immunostaining of mouse brain tissues and MGCs confirmed AK5 expression in microglia, showing co-localization with Iba1-positive cells (Fig. 1E, F).

### AK5 knockdown suppresses NO production and pro-inflammatory cytokine expression in MGCs without inducing cytotoxicity

**Fig. 2:**
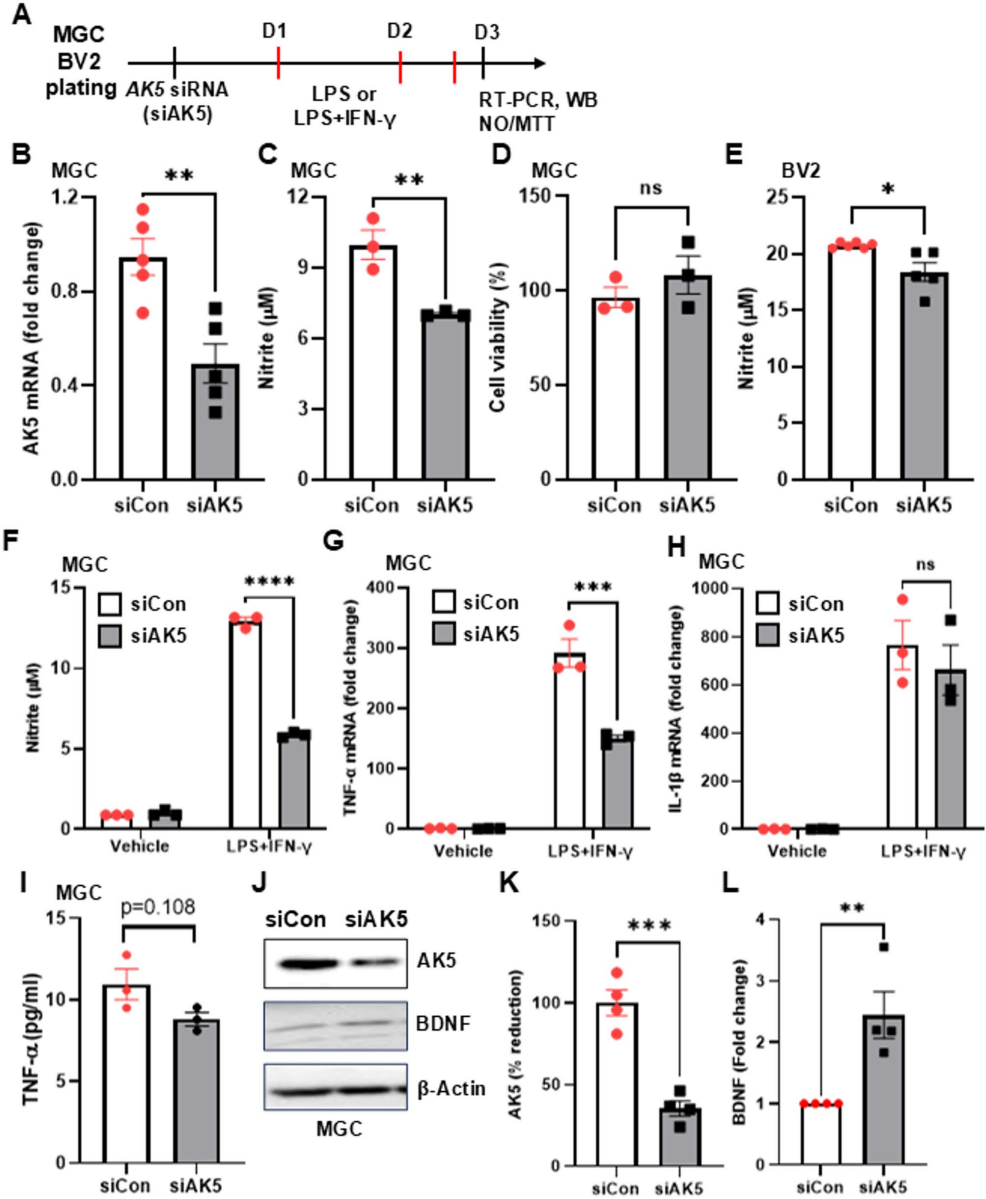
Anti-inflammatory effect of AK5 siRNA in mouse glial cells (MGCs). **A** Experimental timeline. MGCs were transfected with siRNA targeting AK5 (siAK5) or control siRNA (siCon). After 24 h, cells were stimulated with lipopolysaccharide (LPS, 1 μg/mL) for 48 h. Real-time RT-PCR, nitric oxide (NO) assay, and MTT assay were performed 72 h post-transfection. **B** AK5 mRNA production was assessed by real-time RT-PCR. **C** NO production was measured 48 h after LPS stimulation. **D** Cytotoxicity was evaluated by MTT assay 48 h after LPS stimulation. **E** NO production was measured 24 h after LPS (100 ng/mL) stimulation in BV2 cells. **F** NO production was measured after 24 h co-stimulation with LPS (1 μg/mL) and IFN-γ (50 U/mL) in MGC. TNF-α (**G**) and IL-1β (**H**) mRNA expression levels were measured in MGC following stimulation with LPS (1 μg/mL) for 6 h using real-time RT-PCR. **I** TNF-α protein levels in the supernatant of MGCs were measured by ELISA 24 h after treatment with LPS (1 μg/mL) and IFN-γ (50 U/mL). **J** AK5 protein knockdown was assessed by western blot. AK5 (**K**) and BDNF (**L**) protein levels were quantified and normalized to β-actin in MGC lysates. siCon, control siRNA; siAK5, siRNA for AK5 gene knockdown. Data are presented as mean ± SEM. *ns* not significant; *p* > 0.05, *p < 0.05, **p < 0.01, ***p < 0.001, ****p < 0.0001 vs. siCon, determined by unpaired two-tailed Student’s t-test (**B**–**E**, **I**–**L**) or two-way ANOVA with Tukey’s multiple comparisons test (**F**–**H**)

To investigate the role of AK5 in glial inflammatory responses, MGCs were transfected with siRNA targeting AK5 (siAK5) or control siRNA (siCon). As shown in Fig. 2A, after 24 h of siRNA transfection, cells were stimulated with LPS (1 μg/mL) and subsequent analyses were performed 72 h post-transfection. Quantitative RT-PCR analysis confirmed that siAK5 transfection effectively reduced AK5 mRNA levels by more than 50% compared with siCon (Fig. 2B).

LPS-induced NO production, a key marker of glial activation, was significantly reduced in siAK5-transfected MGCs without cytotoxicity (Fig. 2C, D). Similar effects were observed in BV-2 cells (Fig. 2E). Under stronger stimulation with LPS/IFN-γ, siAK5 again suppressed NO production without affecting viability (Fig. 2F). Moreover, AK5 knockdown decreased TNF-α mRNA and modestly reduced IL-1β mRNA (Fig. 2G, H). ELISA confirmed a trend toward lower TNF-α protein levels, though not significant (*p* = 0.108; Fig. 2I). Western blot analysis further confirmed AK5 protein knockdown in MGCs, which was accompanied by a reduction in BDNF protein levels (Fig. 2J, K, L).

### AK5 knockdown enhances microglial phagocytic activity

**Fig. 3:**
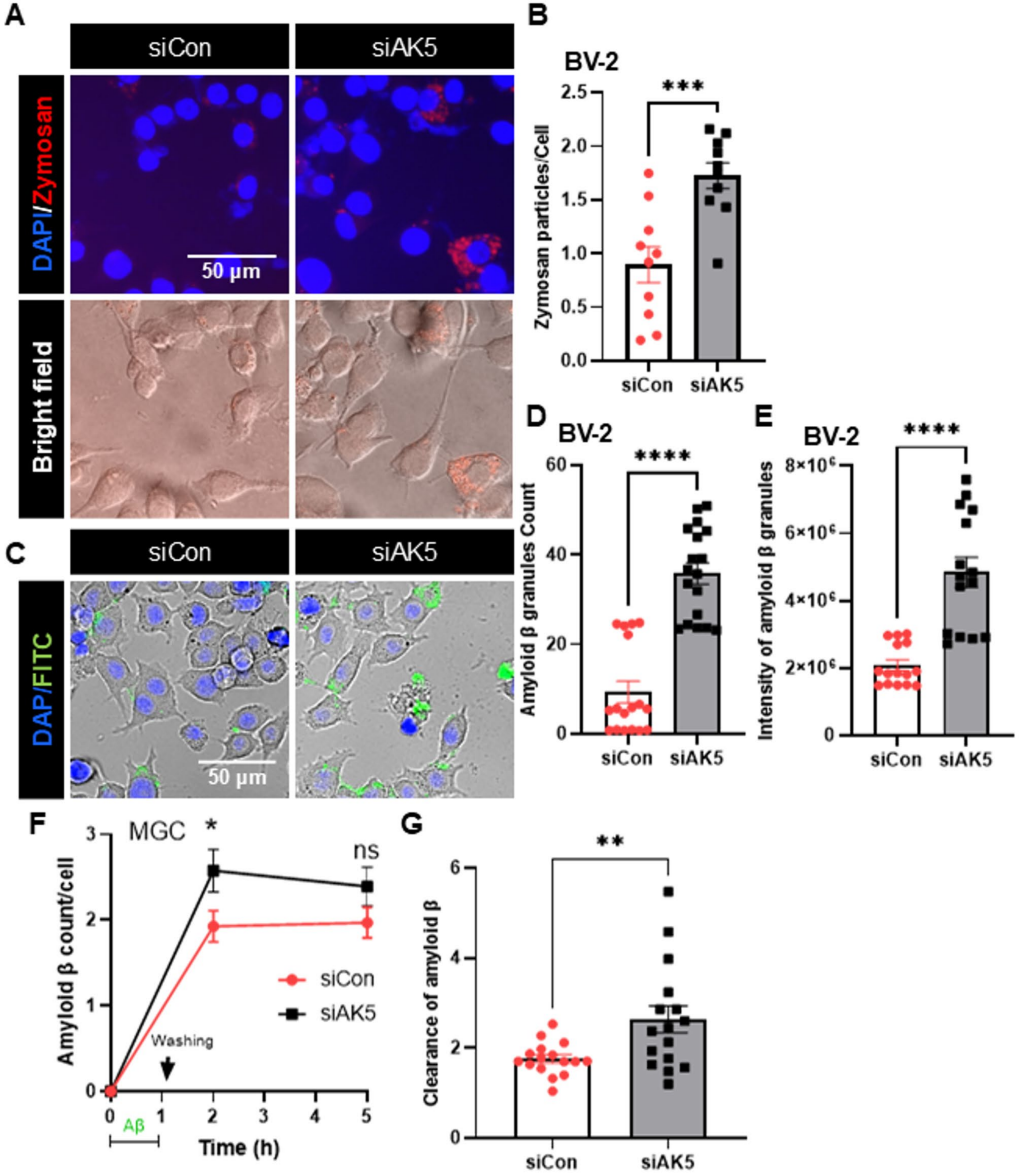
Knockdown of AK5 expression enhances phagocytosis in microglia. **A** BV-2 cells were transfected with siRNA targeting the *AK5* gene, and after 48 h, the cells were incubated with opsonized zymosan red particles for 3 h. **B** Phagocytosed zymosan particles were counted and expressed as the number of zymosan particles per cell. A total of 1499 cells (559 control cells and 940 transfected cells from 10 independent images) were analyzed for quantification. **C** BV-2 cells were cultured in 96-well plates, transfected with siRNA targeting AK5, and incubated for 48 h, followed by incubation with oligomerized FITC-amyloid β (1 µM in serum free DMEM) for 2 h. The phagocytosed FITC-amyloid β was imaged using a fluorescence microscope. **D** Phagocytosed FITC-amyloid β was quantified and expressed as the number of amyloid β granules per cell (**D**) and fluorescence intensity of phagocytosed FITC-amyloid β (**F**). A total of 7200 cells (4336 control cells and 2864 siAK5-transfected cells from 18 images of three independent experiments) were analyzed for quantification. **F** Mouse glial cells (MGCs) were transfected with siRNA targeting the AK5 gene (siAK5) or control siRNA (siCon). After 72 h, cells were incubated with oligomerized FITC-amyloid β (1 µM in serum free DMEM) for 1 h. The phagocytosed FITC-amyloid β was imaged at 1 h and 4 h after washing extracellular amyloid β. **G** Clearance of amyloid β was quantified the reduction of FITC during last 3 h. Internal FITC-amyloid β from 16 images of three independent experiments were analyzed for quantification. Data are presented as means ± SEM. Scale bar, 50 μm. siCon, control siRNA; siAK5, siRNA for *AK5* gene knockdown. *p < 0.05, **p < 0.01, ***p < 0.001, ****p < 0.0001 vs. siCon from unpaired two-tailed Student’s *t-test*

To determine whether AK5 regulates microglial phagocytosis, BV-2 cells were transfected with siAK5 or control siRNA and exposed to opsonized zymosan particles. Microscopy revealed a marked increase in intracellular zymosan particles in siAK5-transfected cells, and quantitative analysis confirmed a significant elevation in phagocytosed particle counts compared with controls (Fig. 3A, B). A similar enhancement was observed with oligomeric Aβ uptake, as both the number of internalized Aβ granules and the fluorescence intensity were significantly higher in siAK5-transfected cells (Fig. 3C–E). Consistent with these findings, primary mouse glial cells (MGCs) with AK5 knockdown also displayed increased uptake of FITC-labeled Aβ and showed accelerated clearance of intracellular Aβ over time, indicating enhanced degradative capacity (Fig. 3F, G). Collectively, these results demonstrate that AK5 depletion enhances the phagocytic capacity of microglia toward both pathogen-associated particles and neurotoxic aggregates.

### AK5 knockdown reduces lipid droplet accumulation in microglia

**Fig. 4:**
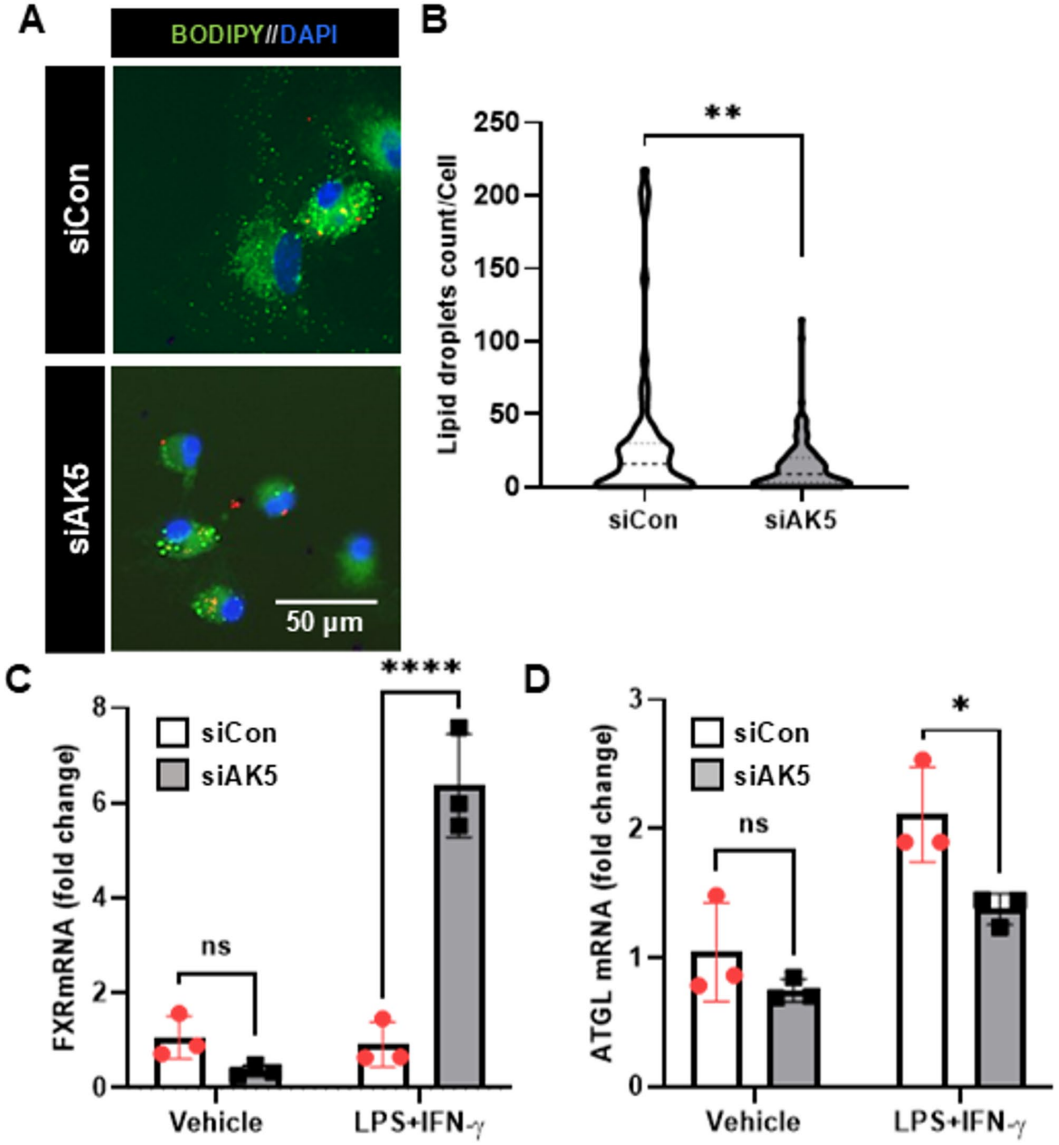
Knockdown of AK5 expression reduces lipid droplet accumulation in microglia. **A** Representative fluorescence images of primary microglial cells transfected with control siRNA (siCon) or AK5-targeting siRNA (siAK5). After 48 h, cells were incubated with opsonized zymosan red particles and BODIPY (green, lipid droplets) and Hoechst (blue, nuclei) for 30 min. **B** Lipid droplet number per cell was quantified from BODIPY-stained images. A total of 182 cells (63 siCon and 119 siAK5-transfected cells) were analyzed. FXR (**C**) and ATGL (**D**) mRNA expression were quantified in MGC by real-time RT-PCR following 6 h stimulation with LPS (1 μg/mL) and IFN-γ (50 U/mL). Expression levels are shown as fold changes relative to siCon. Scale bar = 50 μm. siCon, control siRNA; siAK5, siRNA targeting AK5. Data are presented as mean ± SEM. ns, not significant; *p* > 0.05, *p < 0.05, **p < 0.01, ****p < 0.0001 vs. siCon, determined by unpaired two-tailed Student’s t-test (**B**) or two-way ANOVA with Tukey’s multiple comparisons test (**C**, **D**)

To investigate the role of AK5 in microglial lipid metabolism [25, 26], we first assessed lipid droplet accumulation following AK5 knockdown. Lipid droplets were visualized by BODIPY staining after stimulation with opsonized zymosan particles (Fig. 4A). Quantitative analysis showed a significant reduction in the number of lipid droplets per cell in siAK5-transfected cells compared with controls (*p* < 0.01, Fig. 4B).

Given that Farnesoid X Receptor (FXR) activation suppresses lipid droplet accumulation and modulates microglial inflammatory responses [27, 28], we next examined FXR expression following AK5 knockdown. Quantitative RT-PCR revealed a significant upregulation of FXR mRNA in siAK5-transfected cells compared with controls after LPS and IFN-γ stimulation (*p* < 0.0001, Fig. 4C), To further explore the molecular mechanisms underlying these metabolic changes, we assessed the expression of adipose triglyceride lipase (ATGL), a key enzyme responsible for lipid droplet turnover. Surprisingly, ATGL mRNA levels were significantly decreased in siAK5-transfected cells (Fig. 4D), despite the reduction in lipid droplet content. This paradoxical finding suggests that FXR upregulation may compensate for reduced ATGL expression by promoting alternative lipid turnover pathways and maintaining lipid homeostasis under inflammatory conditions. Collectively, these results indicate that AK5 knockdown alters microglial lipid metabolism through enhanced FXR signaling.

### AK5 SNPs associated with Alzheimer’s disease risk

**Fig. 5:**
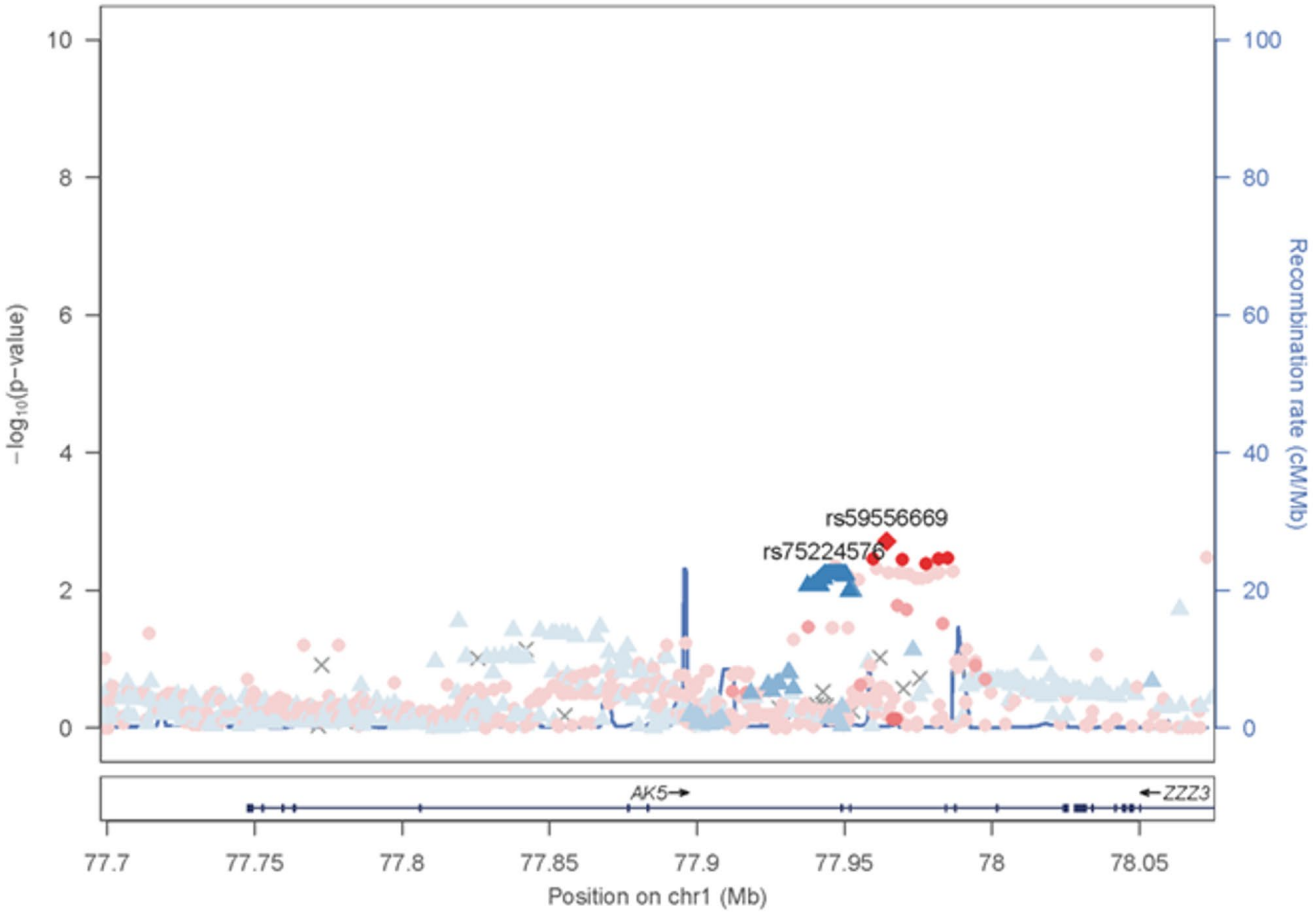
Regional association plot of the AK5 locus highlighting lead SNPs associated with AD. Regional association plot showing genetic variants across a 350 kb window of chromosome 1 (chr1:77.70–78.05 Mb, GRCh38) encompassing the AK5 locus. The x-axis represents the genomic position in Mb, and the left y-axis indicates the − log_10_(*p* value) for the association of each SNP with AD. Higher values on the y-axis correspond to stronger statistical evidence of association. The right y-axis shows the local recombination rate (cM/Mb), plotted as a blue line. The lead SNP rs59556669, which showed the strongest association, is highlighted as red diamond-shaped dot. All other SNPs are represented as circles or triangles, with colors corresponding to the degree of linkage disequilibrium (LD, r^2^) with rs59556669, calculated using the 1000 Genomes Project East Asian (EAS) reference panel (red = high LD, orange/yellow = moderate LD, light blue/blue = low LD)

Previous GWAS have identified 53 SNPs associated with AD risk (*p* < 1 × 10^−4^) [23]. To assess the potential contribution of AK5, we re-analyzed SNPs within or near the AK5 locus in a case–control study (1110 AD and 1172 CN) using logistic regression with age and sex as covariates. A volcano plot revealed 61 SNPs with a false discovery rate below 10% (Fig. 5, Table 3). Among these, rs59556669 and rs75224576 were significantly associated with AD, with odds ratios (OR) of 1.281 (*p* = 1.96 × 10^−3^) and 1.367 (*p* = 5.94 × 10^−3^), respectively. In sex-stratified analyses, rs59556669 showed a stronger association in females (OR 1.312, *p* = 0.0079) than in males (OR 1.247, *p* = 0.085), although effect sizes were comparable.

**Table 3:** Associations of AK5 SNPs with AD risks

SNP	m	M	MAF	OR	SE	L95	U95	*P*	SNP type
rs59556669	C	T	0.183	1.281	0.080	1.095	1.497	0.00196	Intron variant
rs75224576	T	C	0.079	1.367	0.114	1.094	1.709	0.00594	Intron variant
rs79427334	C	T	0.079	1.367	0.114	1.094	1.709	0.00594	Intron variant

*SNP* single nucleotide polymorphism, *M* major allele, *m* minor allele, *MAF* minor allele frequency, *OR* odds ratio, *SE* standard error, *L95* lower bounds of the 95% confidence interval for the odds ratio, *U95* upper bounds of the 95% confidence interval for the odds ratio, *P p* value, *SNP type* type of SNP

The regional association plot (Fig. 5) indicates a concentrated signal around these variants, supporting the involvement of AK5 genetic variants in AD susceptibility. Regional association analysis across a 350 kb window at the AK5 locus (chr1:77.70–78.05 Mb, GRCh38) showed that the lead SNPs lie within a block of variants in strong linkage disequilibrium (LD), calculated using the 1000 Genomes East Asian (EAS) reference panel. A cluster of significant signals was observed in this region of reduced recombination (Fig. 5). Notably, these SNPs were not in linkage disequilibrium with Apolipoprotein E (APOE) loci, suggesting that AK5 may represent an independent genetic risk factor for AD.

### Rare *AK5 variants are associated with brain atrophy* and amyloid-β deposition

**Fig. 6:**
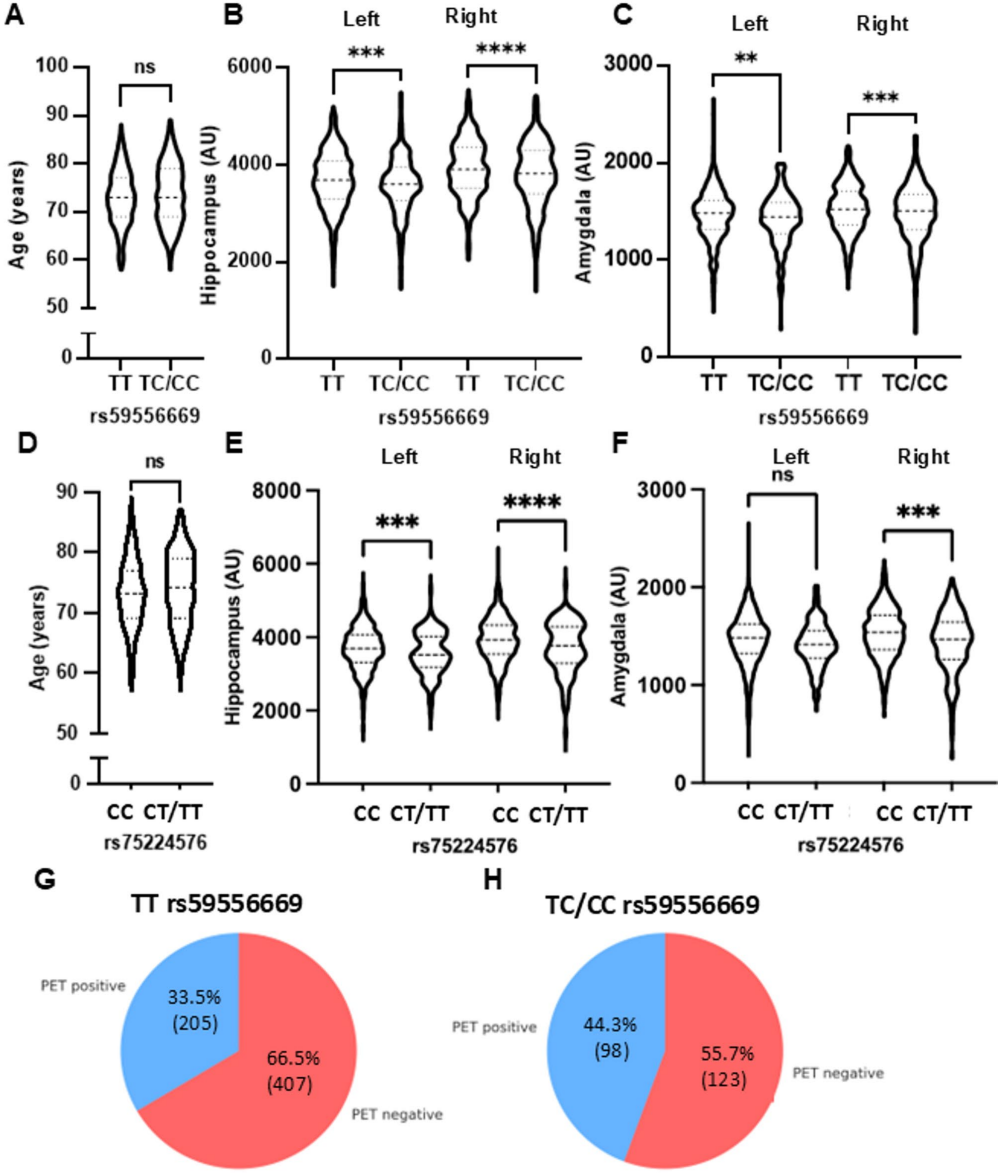
Brain MRI and amyloid PET measures in rs59556669 and rs75224576 minor allele carriers. **A** Violin plots of age distribution in rs59556669 non-carriers TT and carriers TC/CC. **B** Violin plots of hippocampal volume in rs59556669 non-carriers and carriers. **C** Violin plots of amygdala volume in rs59556669 non-carriers and carriers. **D** Violin plots of age distribution in rs75224576 non-carriers CC and carriers CT/TT. **E** Violin plots of hippocampal volume in rs75224576 non-carriers and carriers. **F** Violin plots of amygdala volume in rs75224576 non-carriers and carriers. Data are presented as mean ± SEM. ns, not significant; *p < 0.05, **p < 0.01, ***p < 0.001, ****p < 0.0001 versus TT or CC. Statistical analyses were performed using unpaired two-tailed Student’s t-test (**A**) and (**D**), two-way ANOVA with Tukey’s multiple-comparisons test (**B**–**F**). **G** and **H** Pie charts showing amyloid PET-positive and PET-negative distribution in rs59556669 non-carriers and carriers, with corresponding counts and percentages indicated

To assess the impact of AK5 variants rs59556669 and rs75224576 on brain structural traits and amyloid burden, we analyzed MRI volumetric data and amyloid-PET images. Age distributions did not differ significantly between minor allele carriers (TC/CC) and non-carriers (TT) of rs59556669 or between CC and CT/TT carriers of rs75224576 (Fig. 6A, D), indicating that age was not a confounding factor in structural comparisons. MRI analysis revealed that minor allele carriers of both rs59556669 and rs75224576 exhibited significantly reduced hippocampal and amygdala volumes compared with non-carriers (Fig. 6B, C, E, F). These structural changes were consistent with known patterns of atrophy observed across the clinical continuum from normal cognition to AD, as confirmed in our dataset (Table 2). While standardized uptake value ratio (SUVR) scores from amyloid PET scans did not show statistically significant differences between genotype groups (Supplementary Figs. 1 and 2), the proportion of amyloid PET-positive individuals was significantly higher among carriers of the rs59556669 minor allele. Specifically, 44.3% of minor allele carriers were PET-positive, compared with 33.5% of non-carriers (Fig. 6G, H). This difference was statistically significant (Fisher exact test, *p* = 0.005), suggesting that AK5 genetic variation is associated with increased amyloid deposition.

## Discussion

Our study identifies a functional role for AK5 in microglia and suggests its potential relevance as a therapeutic target for Alzheimer’s disease (AD). AK5 knockdown in microglia reduced pro-inflammatory responses, including decreased nitric oxide production and TNF-α expression, while enhancing phagocytic activity. Furthermore, AK5 knockdown induces metabolic reprogramming, characterized by reduced lipid droplets accumulation and ATGL mRNA levels, along with increased FXR mRNA levels. Notably, genetic analysis revealed that AK5 SNPs rs59556669 and rs75224576 were significantly associated with an increased risk of AD and with reduced volumes of the hippocampal and amygdala.

Targeting overactivated microglia presents a promising strategy for therapeutic intervention against neuroinflammation in AD [29]. Microglia, as the resident macrophages of the central nervous system, play vital roles in immune surveillance, homeostasis, and neuroinflammation [30]. Activated microglia typically assume one of two phenotypes: the pro-inflammatory phenotype, which is deleterious to neurons and is characterized by overproduction of inflammatory cytokines such as TNF-α, IL-6 and IL-1β, or the anti-inflammatory phenotype, which confers regenerative benefits, including the secretion of BDNF and anti-inflammatory cytokines such as IL-4, IL-10, and TGF-β [2, 31], and increased phagocytic functions [32]. Modulating microglial polarization represents a promising approach for mitigating inflammation-related damage. Our findings suggest that AK5 depletion in microglia induces a shift toward an anti-inflammatory phenotype and enhances phagocytic activity, positioning AK5 as a potential target for AD treatment.

Knockdown of AK5 in microglia induced a metabolic shift toward an anti-inflammatory phenotype, characterized by reduced lipid droplet accumulation and increased FXR expression. This metabolic reprogramming highlights the complex regulatory networks underlying neuroinflammation and suggests potential therapeutic avenues for diseases such as AD. Lipid droplets are dynamic organelles central to lipid storage and energy metabolism, and in microglia their accumulation has been linked to a pro-inflammatory state by providing reservoirs of bioactive lipids that fuel inflammatory responses. Consistent with previous reports showing that elevated lipid droplets correlate with microglial activation and pro-inflammatory cytokine production [33, 34], we found that AK5 knockdown reduced both lipid droplets and ATGL expression, thereby promoting an anti-inflammatory phenotype. Notably, pharmacological inhibition of ATGL with atglistatin has also been associated with anti-inflammatory effects [35].

AK5 plays a critical role in adenine nucleotide metabolism and cellular energy balance. Inhibition of AK5 disrupts this balance, potentially leading to decreased AMP production and altered ATP/ADP ratios. This disruption can shift microglia from a pro-inflammatory glycolytic pathway toward oxidative phosphorylation (OXPHOS) and fatty acid oxidation, metabolic pathways typically associated with an anti-inflammatory phenotype. This effect may be attributed to the observed upregulation of FXR expression following AK5 knockdown (Fig. 4), suggesting a regulatory axis where reduced AK5 activity creates metabolic conditions favorable for FXR activation. FXR, a nuclear receptor that regulates lipid and glucose metabolism and exerts anti-inflammatory effects, has been shown to inhibit pro-inflammatory responses in macrophages by promoting oxidative metabolism and reducing dependence on glycolysis [36]. FXR activation can further attenuate neuroinflammation by modulating metabolic pathways that support an anti-inflammatory state. FXR activation significantly inhibits LPS-induced neuroinflammation in microglial cells and is negatively regulated by inflammatory molecules such as cytokines and NF-κB [37, 38]. FXR is negatively regulated by inflammatory molecules such as cytokines and NF-κB [39]. The upregulation of FXR following AK5 knockdown suggests that AK5 may be a key regulator of FXR expression and the subsequent anti-inflammatory response in microglia. Therefore, targeting AK5 not only alters the microglial inflammatory phenotype but also modulates crucial metabolic pathways, providing a multifaceted approach to mitigating neuroinflammatory diseases such as AD.

AKs are vital enzymes involved in cellular energy metabolism and are universally distributed across living organisms. Their relevance extends to pathological conditions, including metabolic syndrome and neurodegenerative disorders [13]. Previous investigations have indicated that adenylate kinase 1 (AK1) expression is significantly increased in brains affected by AD [40] and is induced by Aβ(42) in primary neurons [41]. Ectopic expression of AK1 enhanced tau phosphorylation, while AK1 knockdown reduced Aβ(42)-induced hyperphosphorylation of tau [40], a pivotal process in neurodegenerative diseases such as AD [41]. AK5, a less studied isoform, has not been extensively researched in the context of neurodegenerative diseases. Given the discovery of decreased AK5 expression in the hippocampal region of patients with AD and in brain tissues of TG mice (Fig. 1), we hypothesized that AK5 may play a significant role in AD pathophysiological processes, particularly in microglia.

Exploring AK’s fundamental role is crucial to understanding why we focus on AK5 in microglia. Central to metabolic regulation is the AK family of isoenzymes, which continually monitors cellular adenine nucleotide balance. AK generates AMP signals and facilitates their transmission to various AMP-sensitive components, including those involved in glycolytic and glycogenolytic pathways, as well as metabolic sensors and effectors like AMP-activated protein kinase (AMPK) [42, 43]. Recent studies have revealed that activating AMPK within microglia induces a shift toward an anti-inflammatory phenotype [44, 45]. A decrease in AMP signaling reduces ATP-generating pathways while activating ATP-consuming pathways, resulting in decreased ATP levels within the cell. This reduction in ATP leads to an increase in ADP, which is subsequently converted to AMP via AK [14], maintaining the balance of cellular adenine nucleotides. We hypothesize that knockdown of AK5, a subtype of AK, in microglia initially diminishes AMP signaling, reducing ATP-generating and activating ATP-consuming pathways. This leads to a decline in ATP levels and a rise in ADP levels within the cells. This accumulation of ADP likely triggers the activation of other AK subtypes within microglia, culminating in increased AK activity throughout the cell and an overall amplification of AMP signaling. Ultimately, this elevated AMP signaling enhances AMPK levels, signaling a transition toward an anti-inflammatory microglial phenotype. Chin et al.’s finding that AK4 represses AMPK and cooperates with HIF-1α to boost IL-1β/IL-6/TNF-α expression provides independent support for our AK-to-AMPK immunometabolic model [46]. Although this proposed mechanism remains hypothetical based on our current findings, it provides a compelling direction for future research aimed at elucidating the role of AK5 in microglial energy metabolism and inflammatory regulation.

While our study is the first to elucidate the relationship between AK5 and microglial phenotype, certain limitations remain. We exclusively examined the effects of AK5 knockdown, without addressing the consequences of its overexpression. Future studies employing overexpression systems, such as adeno-associated virus (AAV)-mediated delivery of AK5, are warranted to determine whether increased AK5 expression elicits pro-inflammatory responses in microglia. Moreover, our findings were derived from in vitro experiments using cultured cells, limiting their direct in vivo relevance. To validate our observations in a physiological context, functional studies using animal models with microglia-specific AK5 knockdown or overexpression will be essential. Lastly, as suggested by our MGC data, AK5 may also regulate other glial functions beyond microglia and may additionally influence neuronal functions, which should be explored in future research. Furthermore, the impact of intronic AK5 variants on its expression and function remains to be clarified, ideally through integrative analyses combining single-cell RNA sequencing and GWAS data.

In this study, we observed a reduction in AK5 expression in the hippocampal region of patients with Alzheimer’s disease (AD); however, the underlying cause and timing of this decrease remain unclear. Changes in AK5 expression may be related to age, as AD is a neurodegenerative disease strongly associated with aging. This brings us to the potential role of epigenetic modifications in *AK5* regulation. Epigenetic changes, such as DNA methylation, may influence *AK5* expression. For instance, the hypermethylation of the *AK5* promoter has been reported in certain cancers, leading to reduced AK5 expression [47]. It is possible that similar mechanisms are at play in the aging brain, contributing to the decreased AK5 expression observed in AD. Longitudinal studies using 5xFAD mice or post-mortem tissues will be crucial for tracking changes in AK5 function and expression during AD progression. Despite these limitations, our findings hold significant implications for therapeutic interventions, both in preclinical models and potentially in clinical settings.

In a clinical context, our findings hold promise for informing patient management, particularly in AD cases. The *Apolipoprotein E (APOE)* gene is widely recognized as the most influential and prevalent genetic factor associated with the disease, implicated in over half of all AD cases [48, 49]. Our study found that *AK5* SNPs are not in linkage disequilibrium with the APOE loci, indicating that AK5 can serve as a significant genetic marker independently of *APOE*. Genetic testing for both *APOE* and *AK5*, particularly the rs59556669 and rs75224576 SNPs, may help to refine our understanding of AD susceptibility. In support of its relevance, transcriptomic and metabolomic studies have previously reported that AK5 expression is reduced in the brains of AD patients, and that purine metabolism is disrupted in a stage- and region-specific manner [50]

Future studies in larger cohorts are needed to clarify the compound risk associated with AK5 SNPs in dementia. In particular, their relationship with established AD risk factors such as hyperlipidemia and chronic inflammatory diseases warrants further investigation. Our data show that carriers of AK5 SNPs have higher LDL cholesterol levels than non-carriers (Supplementary Tables 2 and 3), suggesting a potential metabolic link. These findings underscore the value of incorporating AK5 SNP profiling into broader AD risk assessments. Considering such compound risk factors may provide a more integrative understanding of AD susceptibility, especially for genes with modest effect sizes. Moreover, therapeutic strategies targeting AK5 could offer a potential approach to slowing AD progression in the context of metabolic dysfunction.

Our sex-stratified analyses showed that the rs59556669 minor allele was more strongly associated with AD in females, suggesting an interaction between AK5 variants and female-biased metabolic vulnerabilities. Given AK5’s role in energy metabolism, such variants may intensify dyslipidemia and microglial dysfunction, exerting stronger effects in women. Future studies should include sex-specific metabolomic profiling and functional assays to clarify how AK5-dependent metabolism influences AD progression.

*In conclusion*, our study revealed the role of AK5 in microglia, suggesting its potential as a therapeutic target for AD. AK5 knockdown in microglia mitigates inflammatory responses and promotes metabolic reprogramming. Moreover, AK5 SNPs were identified as genetic variants associated with AD. These findings support the consideration of AK5 genetic profiling in AD risk assessment and patient management, thereby opening new avenues for targeted therapies.

## Supplementary Information

Below is the link to the electronic supplementary material.

Supplementary Material 1.

## Data Availability

The datasets analyzed during the current study are available from the corresponding author on reasonable request.

## Abbreviations

AD: Alzheimer's disease
AK5: Adenylate kinase 5
APOE: Apolipoprotein E
ATGL: Adipose triglyceride lipase
DAPI: 4′,6-Diamidino-2-phenylindole
DMEM: Dulbecco’s modified eagle medium
DMSO: Dimethyl sulfoxide
FBS: Fetal bovine serum
FITC: Fluorescein isothiocyanate
FXR: Farnesoid X receptor
GAPDH: Glyceraldehyde 3-phosphate dehydrogenase
GWAS: Genome-wide association study
IFN-γ: Interferon-gamma
IL-1β: Interleukin 1 beta
LPS: Lipopolysaccharide
MGC: Mixed glial cell
MRI: Magnetic resonance imaging
NO: Nitric oxide
PCR: Polymerase chain reaction
PET: Positron emission tomography
RT-PCR: Real time-polymerase chain reaction
SEM: Standard error of the mean
siRNA: Small interfering RNA
SNP: Single nucleotide polymorphism
TNF-α: Tumor necrosis factor alpha
WT: Wild type
