# Supplementary material for: Adenylate kinase 5, a novel genetic risk factor for Alzheimer’s disease, regulates microglial inflammatory activation
Source: Mol Brain. 2025 Nov 26;18:89. doi: 10.1186/s13041-025-01257-z (PMC12659495; doi:10.1186/s13041-025-01257-z)
Supplement: Supplementary file 1 — Supplementary Material 1. [file 13041_2025_1257_MOESM1_ESM.docx]

**Adenylate Kinase 5, a Novel Genetic Risk Factor for Alzheimer’s Disease,**

**Regulates Microglial Inflammatory Activation**

Won Jae Seong^1^, Sang Joon An^2,3^, Jungsoo Gim^4,5,6^, Deepak Prasad Gupta^2^, Junyoung Park^7^, Sarang Kang^4^, Kun Ho Lee^4,5,6^ and Gyun Jee Song^1,2*^

Won Jae Seong: [seongwj11@gmail.com](mailto:seongwj11@gmail.com)

Sang Joon An: [neuroan@ish.ac.kr](mailto:neuroan@ish.ac.kr)

Jungsoo Gim: [jgim@chosun.ac.kr](mailto:jgim@chosun.ac.kr)

Deepak Prasad Gupta: [rasad.gupta@gmail.com](mailto:rasad.gupta@gmail.com)

Junyoung Park: [junyoung0131@gmail.com](mailto:junyoung0131@gmail.com)

Sarang Kang: [csarkd@chosun.kr](mailto:csarkd@chosun.kr)

Kun Ho Lee: [leekho@chosun.ac.kr](mailto:leekho@chosun.ac.kr)

Gyun Jee Song: [gyunjeesong@gmail.com](mailto:gyunjeesong@gmail.com)

^*^Corresponding author

Email: [gyunjeesong@gmail.com](mailto:gyunjeesong@gmail.com); [gjsong@cku.ac.kr](mailto:gjsong@cku.ac.kr)


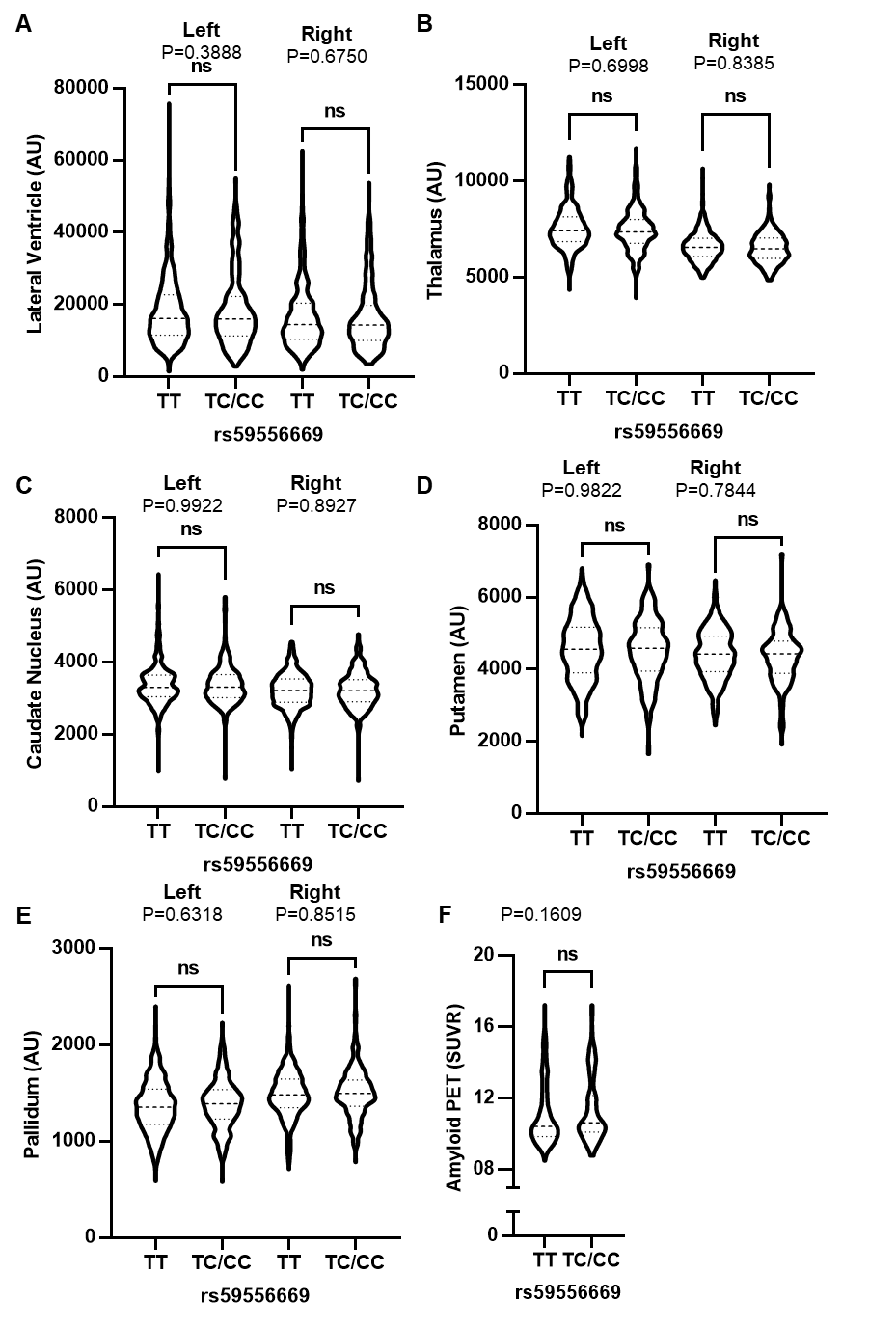


**Supplementary Fig 1.** rs59556669 minor allele carriers showed no difference in the volume of several brain structures and amyloid PET SNUR score. A. Violin plot showing lateral ventricle volume in rs59556669 minorr allele carriers (TC/CC) and non-carriers (TT). B. Violin plot showing thalamus volume. C. Violin plot showing caudate nucleus volume. D. Violin plot showing putamen volume. E. Violin plot showing pallidum volume. F. Violin plot showing amyloid PET SUVR scores. No significant differences were observed in any structure. ns, not significant; p > 0.05 vs. TT from unpaired two-tailed Student’s t-test (F) or two-way ANOVA with Tukey’s multiple-comparisons test (A–E).


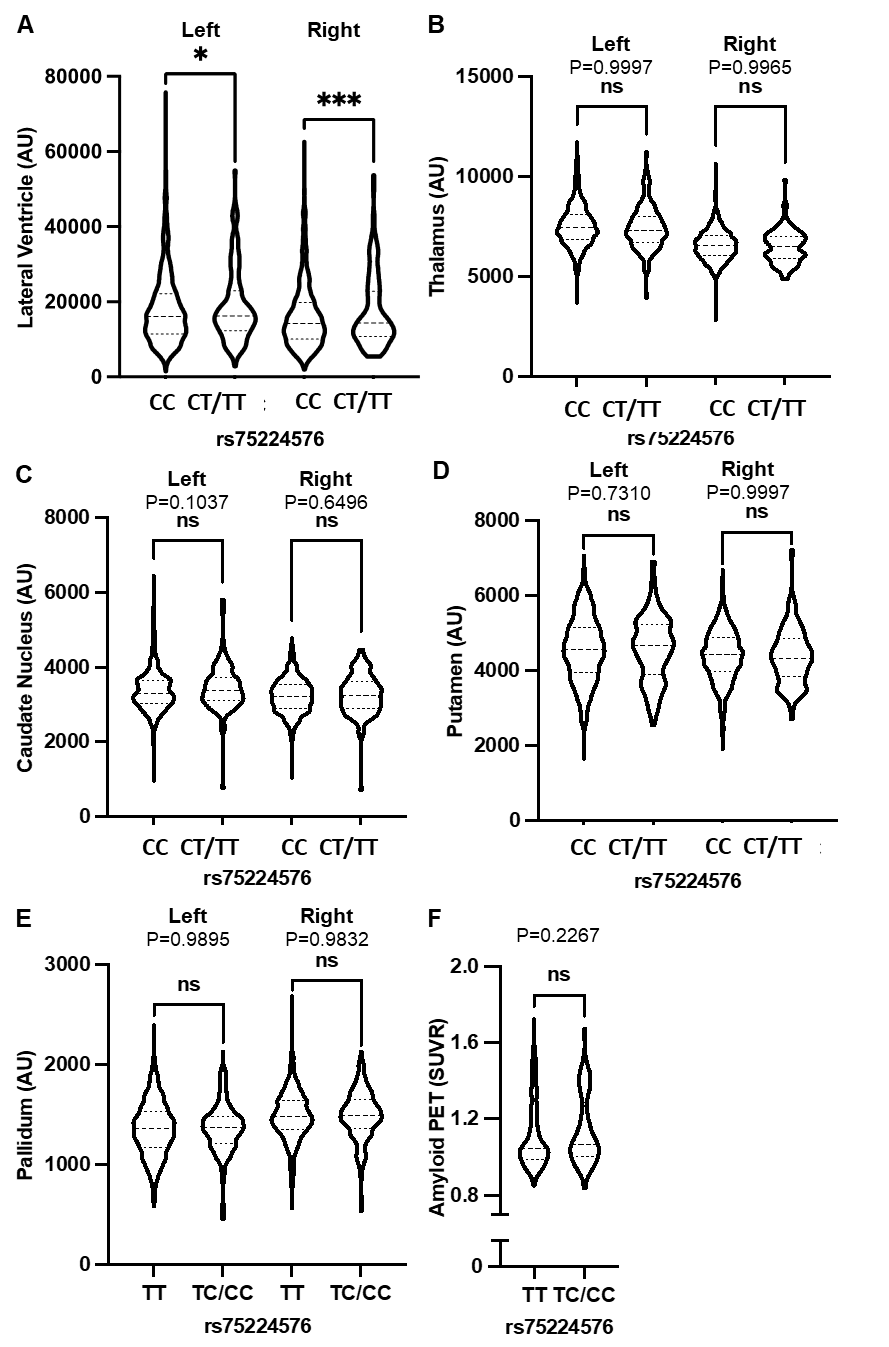


**Supplementary Fig 2.** Effects of rs75224576 major allele on brain volumes and amyloid PET SUVR score. A. Violin plot showing lateral ventricle volume in rs75224576 minor allele carriers (CT/TT) and non-carriers (CC). Carriers showed significantly reduced volume. B. Violin plot showing thalamus volume. C. Violin plot showing caudate nucleus volume. D. Violin plot showing putamen volume. E. Violin plot showing pallidum volume. F. Violin plot showing amyloid PET SUVR scores. No significant difference was detected. ns, not significant; p < 0.05, **p < 0.001 vs. CC from unpaired two-tailed Student’s t-test (F) or two-way ANOVA with Tukey’s multiple-comparisons test (A–E).


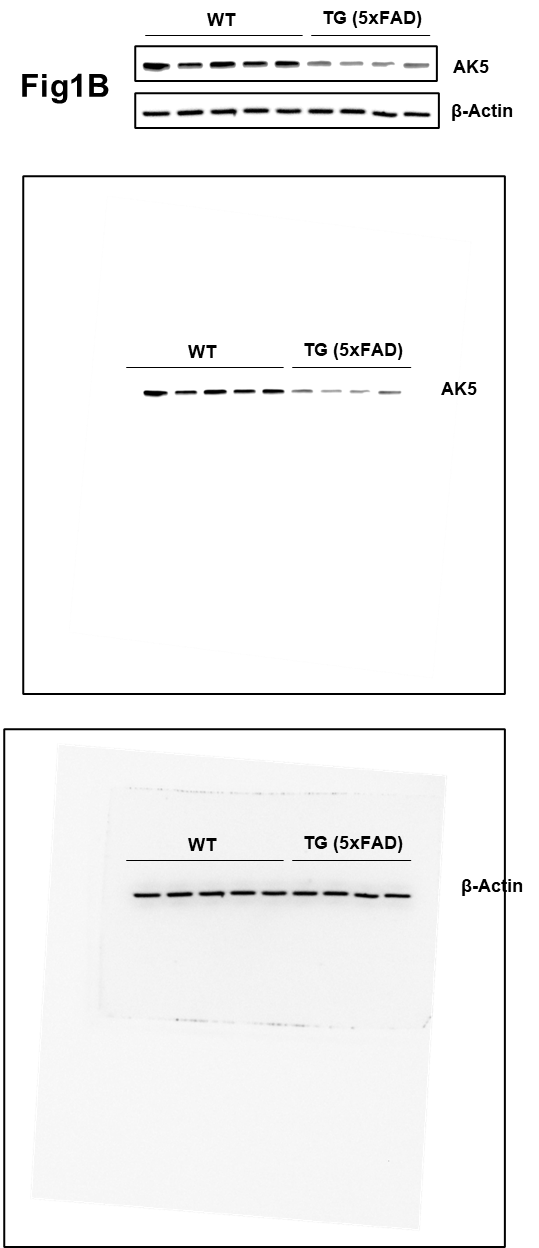


**Supplementary Fig. 3. Full images of Western blot results shown in Fig. 1B.** Full blot images for AK5 and β-actin in cortical tissues of WT and 5xFAD (TG) mice, corresponding to the cropped blots presented in Fig. 1B.

**Supplementary Table 1. Key gene list for wound healing and NO production in glia**

|  | **Migration** | | |  | **NO production** | | |  |
| --- | --- | --- | --- | --- | --- | --- | --- | --- |
| **siRNA** | **P-value** |  | **FC** |  | **P-value** | **FC** | **NO production** | **gene accession number** |
| **Tjp2** | 0.003 |  | 2.05 |  | 0.009 | 0.56 | down | NM_011597 |
| Etnk2 | 0.047 |  | 2.04 |  | 0.001 | 1.53 | up | NM_175443 |
| Pdk4 | 0.000 |  | 2.03 |  | 0.008 | 1.19 | up | NM_013743 |
| Nek5 | 0.001 |  | 1.84 |  | 0.003 | 1.92 | up | NM_177898 |
| Frap1 | 0.000 |  | 1.81 |  | 0.000 | 2.24 | up | NM_020009 |
| Araf | 0.021 |  | 1.81 |  | 0.007 | 1.51 | up | NM_009703 |
| Stk24 | 0.002 |  | 1.79 |  | 0.011 | 1.15 | up | NM_145465 |
| **Csnk1a1** | 0.013 |  | 1.77 |  | 0.008 | 0.57 | down | NM_146087 |
| Alk | 0.005 |  | 1.77 |  | 0.000 | 1.97 | up | NM_007439 |
| Vrk2 | 0.004 |  | 1.73 |  | 0.020 | 1.53 | up | NM_027260 |
| **Ak5** | 0.025 |  | 1.70 |  | 0.041 | 0.76 | down | XM_884305 |
| Oxsr1 | 0.004 |  | 1.70 |  | 0.036 | 2.23 | up | NM_133985 |
| **Map2k1** | 0.038 |  | 1.69 |  | 0.007 | 0.62 | down | NM_008927 |
| Nek3 | 0.045 |  | 1.68 |  | 0.040 | 1.47 | up | NM_011848 |
| **Nrbp2** | 0.004 |  | 1.66 |  | 0.030 | 0.66 | down | NM_144847 |
| Cdc2a | 0.011 |  | 1.65 |  | 0.009 | 1.25 | up | NM_007659 |
| Ulk3 | 0.030 |  | 1.64 |  | 0.000 | 2.51 | up | AC122528 |
| Stk11 | 0.029 |  | 1.64 |  | 0.022 | 1.86 | up | NM_011492 |
| Fn3k | 0.001 |  | 1.62 |  | 0.012 | 1.37 | up | NM_022014 |
| Pdxk | 0.046 |  | 1.59 |  | 0.039 | 3.99 | up | NM_172134 |
| Map3k15 | 0.025 |  | 1.57 |  | 0.046 | 1.28 | up | XM_984997 |
| Pank3 | 0.004 |  | 1.56 |  | 0.000 | 1.99 | up | NM_145962 |
| Stk39 | 0.003 |  | 1.56 |  | 0.027 | 1.55 | up | NM_016866 |
| **Bckdk** | 0.028 |  | 1.56 |  | 0.046 | 0.71 | down | NM_009739 |
| Hck | 0.032 |  | 1.55 |  | 0.027 | 1.43 | up | NM_010407 |
| **Prkar1b** | 0.049 |  | 1.51 |  | 0.017 | 0.74 | down | NM_008923 |
| **Prkch** | 0.045 |  | 1.50 |  | 0.028 | 0.76 | down | NM_008856 |
| Brdt | 0.005 |  | 1.48 |  | 0.025 | 1.15 | up | NM_054054 |
| Prkci | 0.023 |  | 1.48 |  | 0.048 | 1.05 | up | NM_008857 |
| **Mulk** | 0.003 |  | 1.47 |  | 0.048 | 0.64 | down | NM_023538 |
| Nek10 | 0.020 |  | 1.46 |  | 0.002 | 2.15 | up | NM_001034865 |
| Nme7 | 0.003 |  | 1.46 |  | 0.009 | 1.62 | up | NM_178071 |
| Mos | 0.014 |  | 1.45 |  | 0.001 | 1.90 | up | NM_020021 |
| Pgk2 | 0.000 |  | 1.43 |  | 0.017 | 1.93 | up | NM_031190 |
| Alpk3 | 0.018 |  | 1.39 |  | 0.007 | 2.49 | up | NM_054085 |
| Stk35 | 0.032 |  | 1.37 |  | 0.000 | 1.61 | up | NM_001038635 |
| Brsk1 | 0.007 |  | 1.36 |  | 0.006 | 1.20 | up | XM_975912 |
| Mapk6 | 0.006 |  | 1.35 |  | 0.041 | 1.27 | up | NM_015806 |
| Kdr | 0.011 |  | 1.35 |  | 0.006 | 1.72 | up | NM_010612 |
| **Csf1r** | 0.005 |  | 1.33 |  | 0.014 | 0.55 | down | NM_001037859 |
| **Ripk5** | 0.001 |  | 1.33 |  | 0.006 | 0.65 | down | NM_172516 |
| Mknk1 | 0.013 |  | 1.30 |  | 0.045 | 1.44 | up | NM_021461 |
| **BC033915** | 0.020 |  | 1.29 |  | 0.013 | 0.71 | down | XM_916876 |
| **Pip5k1b** | 0.025 |  | 1.28 |  | 0.013 | 0.53 | down | NM_008847 |
| Dcamkl1 | 0.004 |  | 1.26 |  | 0.009 | 1.62 | up | NM_019978 |
| Pkn2 | 0.001 |  | 1.25 |  | 0.002 | 1.97 | up | NM_178654 |
| **Tie1** | 0.026 |  | 1.25 |  | 0.001 | 0.20 | down | NM_011587 |
| Prkag2 | 0.050 |  | 1.23 |  | 0.006 | 1.39 | up | NM_145401 |
| **Bmp2k** | 0.009 |  | 1.20 |  | 0.001 | 0.59 | down | NM_080708 |

P-value from Student’s t-test compared to control siRNA. FC represents fold changes relative to control siRNA.

**Supplementary Table 2. Blood biochemistry panel in the group of rs59556669 minor allele carriers**

| Subject number  (n=835)  M=386:  F=449 | Rs5955666  TT | Rs5955666 CT/CC | P- value |
| --- | --- | --- | --- |
|  | 563 | 248/24=272 |  |
| LDL cholesterol* | 110.695+/-  37.18 | 116.143+/-  40.22 | 0.00377 |
| HDL cholesterol* | 48.51+/-  20.9 | 51.77+/-  11.64 | 0.0048 |
| TG* | 115.7+/-  78.7 | 116.063+/-  75.2 | 0.0071 |
| HBAIC | 5.626+/-  1.55 | 5.6055+/-  1.649 | 0.6927 |
| ASTGOT | 26.33+/-  12.15 | 26.27+/-  15.03 | 0.1782 |
| ASTGPT | 19.64+/-  11.39 | 19.60+/-  12.65 | 0.2289 |

**Supplementary Table 3. Blood biochemistry panel in the group of rs75224576 minor allele carriers**

|  | Rs75224576  CC | Rs75224576  CT/TT | P-value |
| --- | --- | --- | --- |
| Subject number | 738 | 248/24 |  |
| LDL cholesterol* | 110.695+/-  35.46 | 120.00+/-  42.24 | 0.01921 |
| HDL cholesterol | 51.81+/-  16.03 | 51.72+/-  12.68 | 0.9570 |
| TG | 123.86+/-  76.2 | 138.15+/-  70.65 | 0.0942 |
| HBAIC | 5.937+/-  0.93 | 5.953+/-  1.098 | 0.8849 |
| ASTGOT | 27.04+/-  13.12 | 28.52+/-  10.41 | 0.2866 |
| ASTGPT | 20.14+/-  11.60 | 21.66+/-  1.89 | 0.2285 |
